# Gate Modulation of the Spin-orbit Interaction in Bilayer Graphene Encapsulated by WS_2_ films

**DOI:** 10.1038/s41598-018-21787-y

**Published:** 2018-02-21

**Authors:** Amir Muhammad Afzal, Muhammad Farooq Khan, Ghazanfar Nazir, Ghulam Dastgeer, Sikandar Aftab, Imtisal Akhtar, Yongho Seo, Jonghwa Eom

**Affiliations:** 1 Department of Physics & Astronomy and Graphene Research Institute, Sejong University, Seoul, 05006 Korea; 2 Department of Nanotechnology & Advanced Materials Engineering, Sejong University, Seoul, 05006 Korea

## Abstract

Graphene has gigantic potential in the development of advanced spintronic devices. The interfacial interactions of graphene with semiconducting transition metal dichalcogenides improve the electronic properties drastically, making it an intriguing candidate for spintronic applications. Here, we fabricated bilayer graphene encapsulated by WS_2_ layers to exploit the interface-induced spin-orbit interaction (SOI). We designed a dual gated device, where the SOI is tuned by gate voltages. The strength of induced SOI in the bilayer graphene is dramatically elevated, which leads to a strong weak antilocalization (WAL) effect at low temperature. The quantitative analysis of WAL demonstrates that the spin relaxation time is 10 times smaller than in bilayer graphene on conventional substrates. To support these results, we also examined Shubnikov-de Haas (SdH) oscillations, which give unambiguous evidence of the zero-field spin-splitting in our bilayer graphene. The spin-orbit coupling constants estimated by two different measurements (i.e., the WAL effect and SdH oscillations) show close values as a function of gate voltage, supporting the self-consistency of this study’s experimental results. The gate modulation of the SOI in bilayer graphene encapsulated by WS_2_ films establishes a novel way to explore the manipulation of spin-dependent transport through an electric field.

## Introduction

The evolution of graphene, which has a honey-comb structure, has stimulated the exploration of various two-dimensional materials, such as transition metal dichalcogenides (TMDs)^1,2^. Most thin-film TMD crystals are semiconductors and own a particular band gap energy, whereas graphene is a gapless semimetal^3^. Graphene is considered to be a two-dimensional (2D) platform of massless charge carriers due to the Dirac cone nature of the band structure and the existence of two valleys^4,5^. Meanwhile, van der Waals (vdW) heterostructure composed of the vertical stacking of different 2D materials has been developed recently as a compact system. This system provides a new paragon for engineering electronic and spintronic tuneable parameters^6^. Hence, the spin-orbit interaction (SOI) in graphene is focused on theoretically at length but is less explored and investigated experimentally^7–10^. In previous reports, SOI has been enhanced in many ways like chemical doping and conversion of sp^2^ to sp^3^ bonds, which introduce disorders in the electronic structure and charge carrier mobility^7,11^. In addition, the decoration of heavy metal adatoms such as indium (In), thallium (Ti), Iridium (Ir), or gold (Au) on the graphene surface has been proposed to enhance the SOI. All of these methods lead to disorder in the transport quality and a number of limitations for spin transport characteristics, making it difficult to control the SOI^12–15^. The vertically assembled heterostructure of 2D materials has proclivity to tailor the interfacial interaction at the atomic level by shielding the basic structure and integrity of individual layers^16–18^. The heterostructures of graphene with 2D semiconducting TMDs with large band gap appear to be an auspicious factor because of the strong SOI in TMDs. The Dirac nature of electrons in semi-metal graphene demonstrates a huge proximity with SOI without compromising the electronic and semimetal nature of the system. Thus, experimental studies of SOI in graphene-based systems under the influence of the bottom substrate have been reported^19–21^. In previous studies, Avsar *et al*. proposed that the SOI is attributed to the intrinsic defects in WS_2_ substrate, which gives rise to the spin Hall effect at room temperature due to band structure modification^22^. On other hand, Wang *et al*. claimed that the strong SOI originates from the interfacial effect of graphene and WS_2_^21^. A consensus has not been made yet to find the actual nature of SOI in graphene on WS_2_.

Here, we develop an innovative dual gate WS_2_/bilayer graphene/WS_2_ sandwich device to address the gate modulation of SOI by measuring the quantum interference transport and Shubnikov-de Haas (SdH) oscillations. The bilayer graphene (BLG) sandwiched between WS_2_ films demonstrates a prominent and robust phenomenon of weak anti-localization (WAL) at low temperature. The WAL effect in the 2D system is a quantum interference phenomenon, which has assisted for a long time as a direct and precise method to probe the SOI in conductors^23,24^. To analyze the enhancement in the magnitude of SOI in BLG quantitatively, we use the theory of WAL for graphene to fit our magneto-conductivity. The giant SOI in our graphene device is due to the interfacial interaction of WS_2_ on both sides of graphene. This paper demonstrates that the magnitude of SOI relaxation time (*τ*_*so*_) in WS_2_-encapsulated BLG is 10 times smaller than *τ*_*so*_ in graphene on ordinary substrate. It is found that the SOI of the bilayer graphene is tuned by applying gate voltages. To endorse these results, we have also measured SdH oscillations, which provide unambiguous evidence of the zero-field spin-splitting due to a strong SOI. There are two ways to estimate the magnitude of SOI in this study’s system in the framework of the Rashba SOI mechanism. The estimated values of SOI through WAL analysis and SdH oscillation analysis give close results, supporting the self-consistency of this study’s experimental results. The effective gate modification of SOI strength in the graphene-based system enables this study to explore new areas of the field-effect spin transport phenomenon.

## Results

### Characterization of the WS_2_/BLG/WS_2_ sandwich device

**Figure 1:**
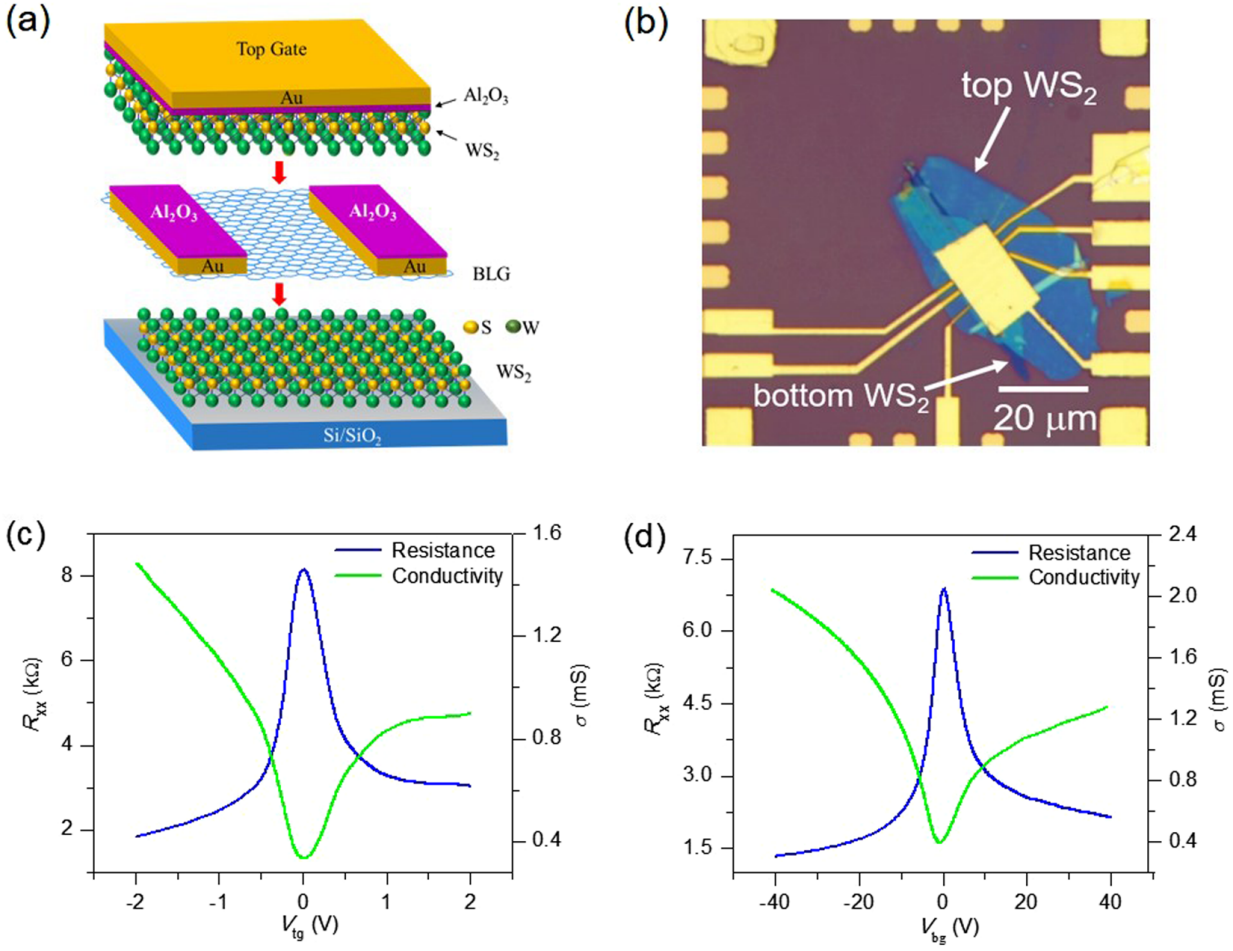
Schematic and electrical characteristics of a WS_2_/BLG/WS_2_ sandwich device. (**a**) Bilayer graphene (BLG) is sandwiched between multilayer WS_2_. (**b**) Optical microscope image of the WS_2_/BLG/WS_2_ sandwich device. (**c**) Resistance and conductivity as a function of top gate voltage (V_tg_). (**d**) Resistance and conductivity as a function of back gate voltage (V_bg_). Measurements were performed in vacuum at T= 4.2 K.

Figure 1(a) shows a schematic of the WS_2_/BLG/WS_2_ sandwich device in which BLG is sandwiched between WS_2_. Figure 1(b) shows an optical image of the final device in a Hall bar configuration. Figure S1(a) shows WS_2_ flake on SiO_2_ (300 nm) with a highly doped Si wafer by the mechanical exfoliation method. The Raman spectra of multilayer WS_2_ on SiO_2_ and on Gr are shown in Fig. S2(a). The ${{\rm{E}}}_{2g}^{1}$ and A_1_ peaks appear at 351 cm^−1^ and 418 cm^−1^, respectively^22^. The Raman spectra of WS_2_ films are almost the same on either BLG or SiO_2_ substrate. Fig. S1(b) shows the bilayer graphene on WS_2_ flake. The Raman G and 2D peaks of BLG appear around 1587 cm^−1^ and 2685 cm^−1^ as shown in Fig. S2(b). The ratio of intensities of G and 2D peaks (I_2D_/I_G_) is ~1.2, which is in agreement with a previously reported value of BLG^25,26^. The thickness is further confirmed by atomic force microscopy (AFM). Fig. S2(c) shows the height profile of WS_2_ on SiO_2_, with the thickness of WS_2_ being ~7 nm. Fig. S2(d) represents the height profile of BLG on WS_2_, with the thickness of BLG being ~0.8 nm.

Figure 1(c) shows resistance as a function of V_tg_. The top gate voltage is swept from −2 V to +2 V, and resistance is measured. Resistance is also measured as a function of the back gate voltage V_bg_ as shown in Fig. 1(d). In both cases, the charge neutrality point (i.e., the Dirac point) lies nearly at zero voltage. The mobility can be calculated by (1/C_g_)(∂σ/∂V), where C_g_ is the gate capacitance. The mobilities calculated by using V_tg_ and V_bg_ are 20,000 cm^2^/V.s and 18,100 cm^2^/V.s, respectively^20,27–29^. When the gate voltages (V_tg_ or V_bg_) are increased from V_tg_ = −2 V and V_bg_ = −40 V, the conductivity of the WS_2_/BLG/WS_2_ sandwich device gradually decreases until V_tg,_ and V_bg_ reach the charge neutrality point. However, conductivity is asymmetric with respect to the Dirac point. The conductivity becomes saturated when both V_tg_ and V_bg_ are increased to positive voltages. The saturation of the conductivity is attributed to the fact that WS_2_ is an n-type semiconductor and starts to conduct at positive gate voltages. At gate voltages larger than the threshold voltage of WS_2_, electrons accumulate at the surface of WS_2_ films and screen the influence of gate electric fields. Because the charge carrier mobility in WS_2_ is much smaller than in BLG, the carriers in WS_2_ give a negligible contribution to transport but behave as an influential source for charge carriers.

**Figure 2:**
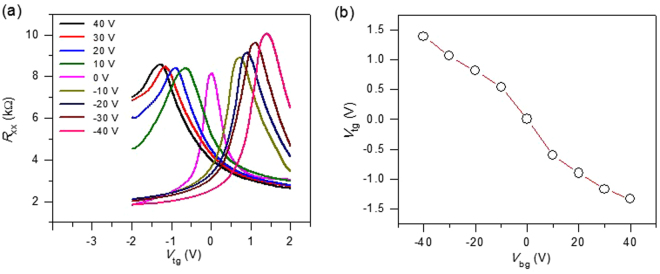
Resistance of the WS_2_/BLG/WS_2_ sandwich device as a function of top gate voltage (V_tg_) at different fixed back gate voltages (V_bg_). (**a**) The different traces are taken with 10-V steps in V_bg_ from 40 V to −40 V. (**b**) The relation between V_tg_ and V_bg_, where the charge neutrality point of bilayer graphene in our device occurs. Measurements were performed in vacuum at T= 4.2 K.

We characterize the basic electrical transport properties of WS_2_/BLG/WS_2_ sandwich devices. In Fig. 2(a), the electrical resistance of device as a function of the top gate voltage V_tg_ at a different fixed back gate voltage V_bg_ is traced. Each trace is taken with 10 V steps in V_bg_ from 40 V to −40 V. The resistance of each trace shows a peak at different charge neutrality points because the total electric field differs. Figure 2(b) shows the relation between V_tg_ and V_bg_, which results in the charge neutrality point of BLG. The linear-like relation indicates a proper function of the dual gates. The slope of the linear dependence is determined by the thickness ratio and permittivity of the top and bottom gate materials^29^.

We also measured the resistance of the WS_2_/BLG/WS_2_ sandwich device as a function of gate voltages in the magnetic field of 9 T at a temperature of 4.2 K. Fig. S4(a) shows the longitudinal (ρ_xx_) and Hall resistivity (ρ_xy_) as a function of V_bg_ with V_tg_ = 0 V. Fig. S4(b) shows the variation in longitudinal (ρ_xx_) and Hall resistivity (ρ_xy_) as a function of V_tg_ with V_bg_ = 0 V. While ρ_xx_ oscillates as the gate voltage passes over the Landau levels, ρ_xy_ shows a plateau when ρ_xx_ becomes a local minimum. However, the ρ_xx_ oscillations and ρ_xy_ plateaus do not appear for positive gate voltages because the conductive WS_2_ layer screens out the electrical fields from the gates. Figure S4(c) shows the Hall conductance (σ_xy_) as a function of V_bg_ with V_tg_ = 0 V. Figure S4(d) shows the Hall conductance with respect to V_tg_ with V_bg_ = 0 V. Under the perpendicular magnetic field, the Hall conductance plateaus of BLG satisfy *σ*_*xy*_ = 4*Ne*^2^/*h*, where N is an integer^22,30–32^ as seen in Fig. S4(c) and (d).

### Weak anti-localization measurements

**Figure 3:**
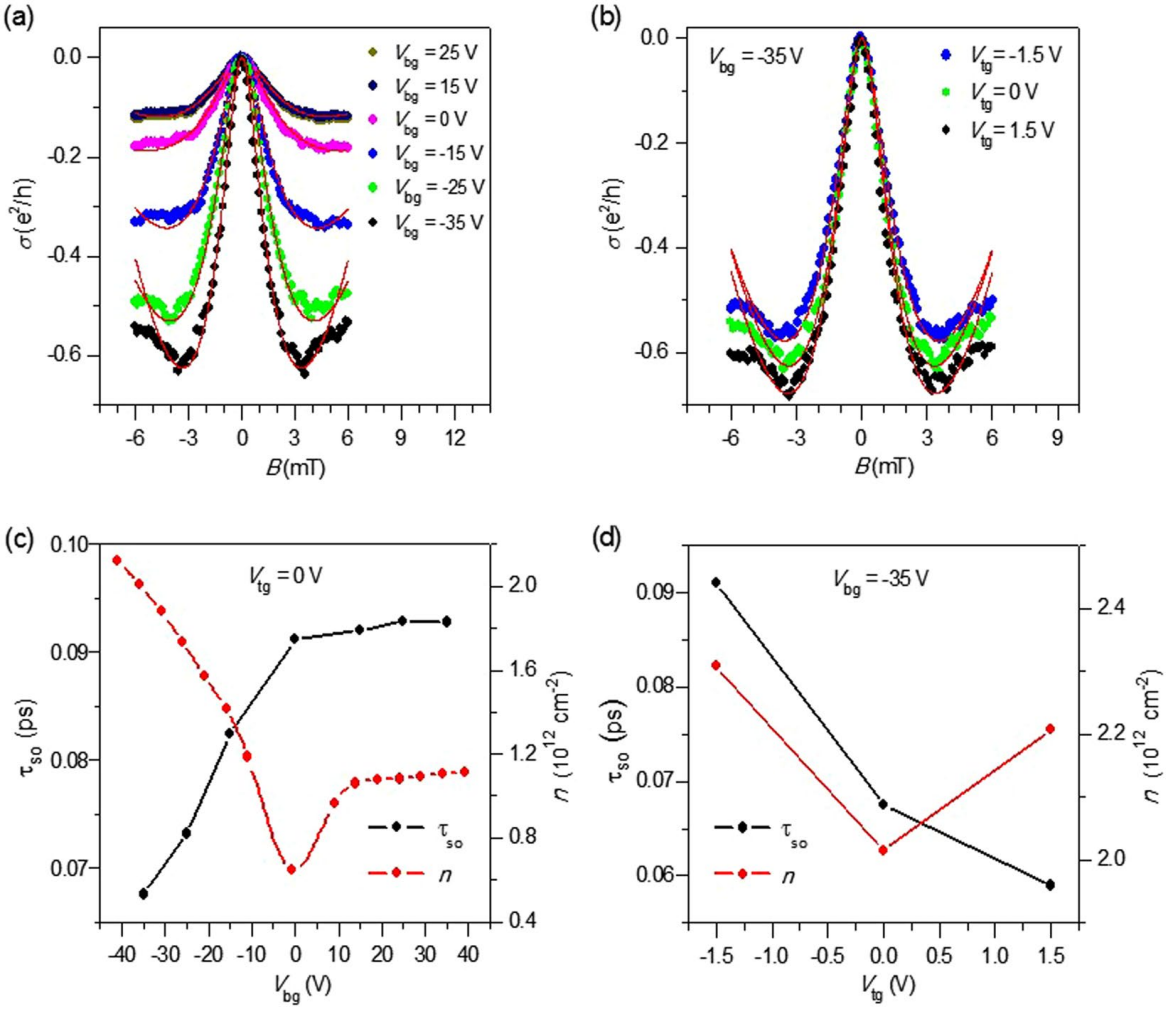
Weak antilocalization measurement. (**a**) Magnetoconductivity (∆σ = σ(B≠0) − σ(B=0) at different back gate voltages (V_bg_). The top gate voltage (V_tg_) is fixed at 0 V. (**b**) Magnetoconductivity with the dual gates applied. (**c**) Spin relaxation time as a function of V_bg_ (black line). The red line is the carrier concentration as a function of V_bg_. (**d**) Spin relaxation time and charge carrier density as a function of V_tg_ at a fixed back gate voltage of −35 V. Measurements were performed in vacuum at T= 4.2 K.

To examine the SOI in WS_2_/BLG/WS_2_ sandwich devices, we measure WAL at a low temperature, which usually demonstrates itself as a distinguishing sharp magneto-conductivity peak at B = 0 T. Figure 3(a) shows the conductivity as a function of magnetic field (B) at V_tg_ = 0 V and different V_bg_’s. Figure 3(b) shows the conductivity of the WS_2_/BLG/WS_2_ sandwich device as a function of B under transverse electric field applied by dual gates. The data are taken at three different combinations of V_tg_ and V_bg_ at T = 4.2 K. Negative magnetoconductivity (∆σ = σ (B ≠ 0) − σ (B = 0) is directly related to WAL. ∆σ reaches the largest value of approximately 0.65 e^2^/h at V_bg_ = −35 V and V_tg_ = 0 V. This type of phenomenon was not observed in single layer graphene (SLG) on ordinary substrates such as SiO_2_, hexagonal boron nitride (hBN), and GaAs^20,33,34^. However, the WAL effect due to the π Berry phase in SLG can be restored if chirality symmetry is preserved in the absence of intravalley scattering. On the other hand, in BLG on ordinary substrate, the electron wave function acquires a 2π Berry phase on back-scattering, which does not give rise to the WAL effect. However, SLG and BLG exhibit the WAL effect on WS_2_ substrate, which provides direct and unambiguous evidence to demonstrate the existence of interface-induced SOI^21,28^.

To analyze the pronounced peak of Δσ around B = 0, we use the WAL theory in graphene, which takes into account the effect of both symmetric and asymmetric SOI terms in bilayer graphene at low temperature^35^.

1 $${\rm{\Delta }}{\rm{\sigma }}\,({\rm{B}})=-\frac{{e}^{2}}{2\pi h}\,[F\,(\frac{{\tau }_{B}^{-1}}{{\tau }_{\phi }^{-1}})-F\,(\frac{{\tau }_{B}^{-1}}{{\tau }_{\phi }^{-1\,}+2{\tau }_{asy}^{-1}})-2F\,(\frac{{\tau }_{B}^{-1}}{{\tau }_{\phi }^{-1\,}+{\tau }_{so}^{-1}})\,].$$

Here, F(x) = ln (x) + Ψ (1/2 + 1/x), where Ψ (x) is the digamma function, ${\tau }_{B}^{-1}=\frac{4DeB}{{\hbar }}\,$, D is the charge carrier diffusion constant, and ${\tau }_{\phi }^{-1}$ is the dephasing rate. ${\tau }_{asy}^{-1}$ describes the asymmetric spin relaxation rate due to the SOI term, which breaks the inversion symmetry in the direction normal to the graphene plane. The total spin relaxation rate is given by ${\tau }_{so}^{-1}=\,{\tau }_{sym}^{-1}+\,{\tau }_{asy}^{-1}$. Figure 3(c) shows the spin relaxation time (*τ*_*so*_) as a function of V_bg_, which is obtained by fitting the parameter of Δσ around B = 0 T in Fig. 3(a) using Equation (1). The red lines in Fig. 3(a) show the fitting lines to the WAL theory. These values of *τ*_*so*_ are much smaller than previously reported values for graphene on SiO_2_, which range from 100 ps to 1 ns, and those for graphene on TMDs^21,36,37^_._ The value of *τ*_*so*_ is 0.0675 *ps* at V_bg_ = −35 V and V_tg_ = 0 V, and it increases monotonically as V_bg_ increases from −35 V to +35 V. However, the charge carrier density (*n*) does not change monotonically due to the ambipolar charcteristics of the graphene field-effect transistor. The red line in Fig. 3(c) represents the charge carrier density as a function of V_bg_. We note that *τ*_*so*_ is changed effectively by the gate voltage but is rather irrelevant to the charge carrier density. We also use the WAL theory to fit the magnetoresistance under dual gate voltages. The red lines in Fig. 3(b) are the fitting lines to the WAL theory at different V_tg_’s at V_bg_ = −35 V. Figure 3(d) shows *τ*_*so*_ and *n* at different V_tg_’s at V_bg_ = −35 V. *τ*_*so*_ decreases from 0.091 ps to 0.059 ps as V_tg_ increases from −1.5 V to 1.5 V. The gate voltage dependence of *τ*_*so*_ in Fig. 3(c) and (d) indicates that the electric field toward the bottom direction increases the SOI in our system.

### Shubnikov-de Haas oscillations

In structures like our sandwich device, a strong local electric field (*E*_*z*_) is generated by the accumulation of electrons in the interfaces with WS_2_ on both sides of the BLG. This electric field acts perpendicular to the motion of electrons. It is predicted that the coupling of electron spin to this local field generates a Rashba type SOI. This type of SOI is described by the Rashba Hamiltonian, ${H}_{R}=\,\alpha \,(\overrightarrow{\sigma }\,\times \,{\overrightarrow{k}}_{F}\,)\cdot \,\overrightarrow{z}$ where ${\overrightarrow{k}}_{F}\,$ represents the electron wave vector, $\overrightarrow{\sigma }$ are the Pauli matrices, and $\overrightarrow{z}$ is a unit vector that is perpendicular to the interface^38^. The crucial parameter *α* represents the strength of SOI, and it is directly proportional (*α* ∝ *E*_*z*_) to the interfacial electric field *E*_*z*_. Thus, in our case, the interface on both sides of BLG enhances the strength of SOI.

**Figure 4:**
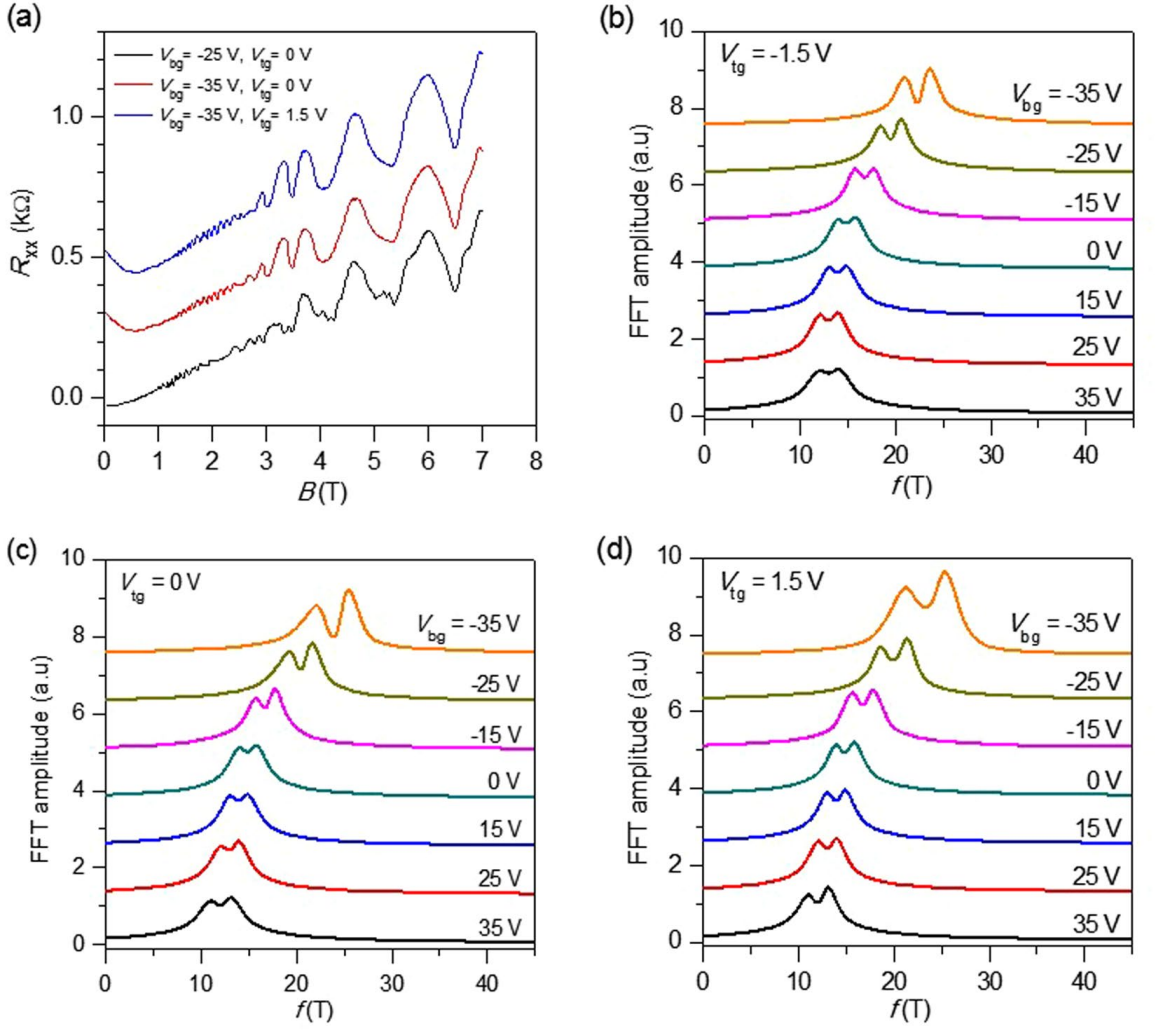
Shubnikov-de Hass (SdH) oscillations with dual gate voltages. (**a**) Shubnikov-de Hass oscillations with dual gate voltages applied at T = 4.2 K. (**b**) Fast Fourier transformation (FFT) amplitude at different V_bg_ with V_tg_ = −1.5 V. (**c**) FFT amplitudes at different V_bg_ with V_tg_ = 0 V. (**d**) FFT amplitudes at different V_bg_ with V_tg_ = 1.5 V.

To analyze the estimated value of SOI in the encapsulated BLG, we measured the Shubnikov-de Haas oscillations. Figure 4(a) shows the effect of V_bg_ and V_tg_ on SdH oscillations at T = 4.2 K. We observed that the beating pattern becomes more prominent and visible by applying high gate voltages. Figure 4(b–d) show the fast Fourier transformations (FFT) of SdH oscillations (R_xx_ as a function of 1/B) at different gate voltages. The oscillation frequency shows a clear dependence on applied gate voltages. The position of peaks in FFT is related to the charge carrier density, which is modified by the applied gate voltages. While the peak position changes rapidly as V_bg_ changes from −35 to 0 V, the amount of change is small for V_bg_ > 0. This is because the screening of the electric field is turned on when the WS_2_ layer becomes a conductor for V_bg_ > 0, consistent with the observations in Fig. 3(c). A pair of peaks in FFT as clear evidence of zero-field spin-splitting due to the induced SOI in our device is witnessed. Two peaks in the FFT of SdH oscillations are clearly seen at all V_bg_’s at different V_tg_’s (see Fig. 4(b–d)). The separation of SdH oscillation frequencies is directly proportional to the area of the splitting of two Fermi surfaces. Therefore, the two peaks in FFT are direct confirmations of the SOI-induced spin-splitting of the Fermi surface of BLG on WS_2_. The enhancement in the magnitude of the frequency splitting that is detected upon varying the charge carrier density points out that the prevailing contribution to the induced SOI is Rashba type.

## Discussion and Conclusion

**Figure 5:**
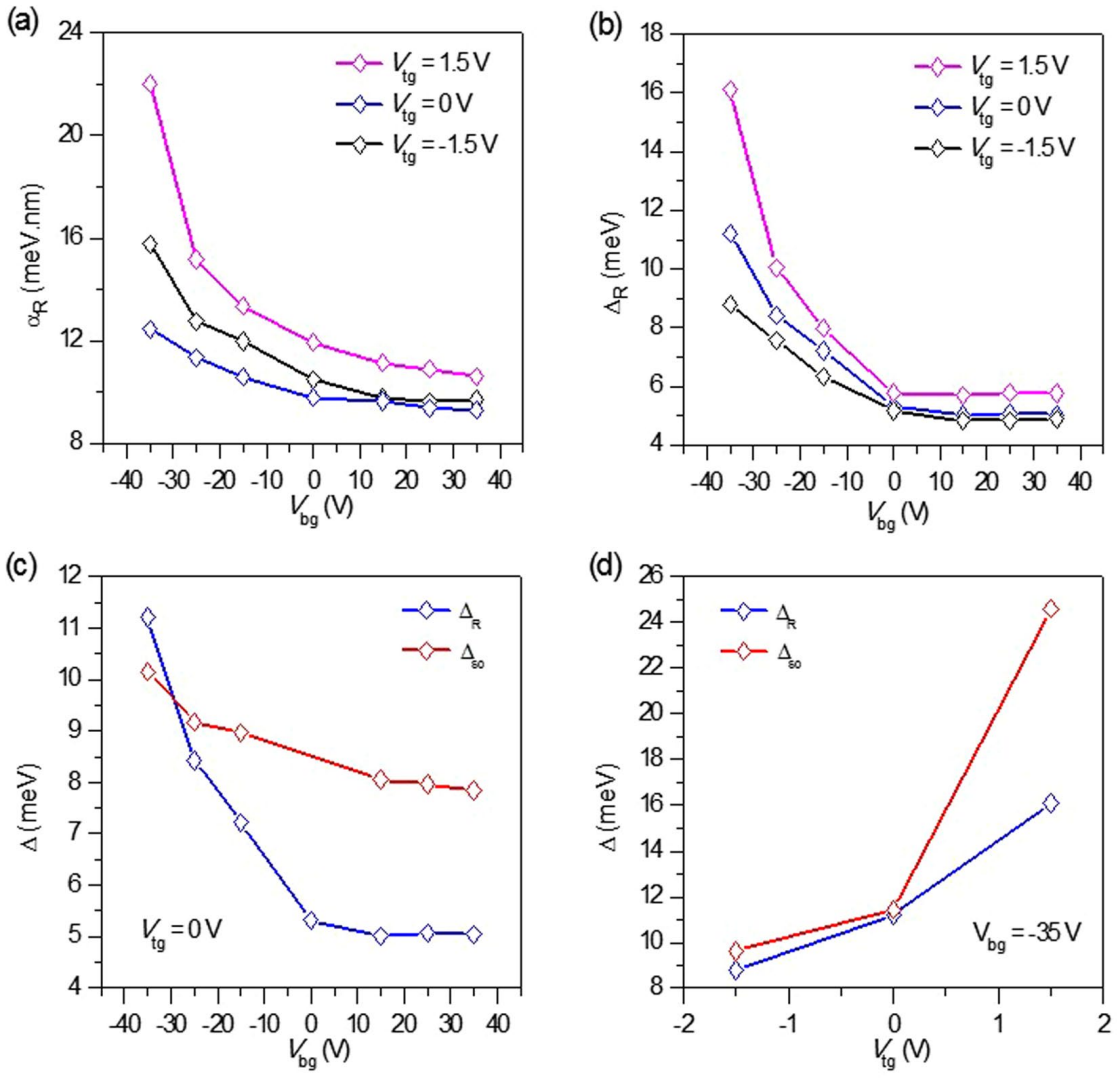
(**a**) Spin-orbital coupling constant (*α*_*R*_) as a function of V_bg_. (**b**) Rashba spin-splitting (${{\rm{\Delta }}}_{R}$) as a function of V_bg_. (**c**) Comparison of ${{\rm{\Delta }}}_{R}$ and ${{\rm{\Delta }}}_{SO}$ as a function of V_bg_ and at V_tg_ = 0 V. (**d**) Dependence of ${{\rm{\Delta }}}_{R}$ and ${{\rm{\Delta }}}_{SO}$ on V_tg_ at a fixed back gate voltage of −35 V. Measurements were performed in vacuum at T= 4.2 K.

The zero-field spin-splitting (Δ_R_) due to Rashba type SOI is given by the relation,${{\rm{\Delta }}}_{R}=\frac{{h}^{2}}{2\pi {m}^{\ast }}({n}_{\uparrow }-{n}_{\downarrow })=$ $\,\frac{e\,h}{2\pi {m}^{\ast }}({f}_{\uparrow }-{f}_{\downarrow })$, where h is the Planck constant and m^*^ is an effective mass of electrons and holes in bilayer grapheme^39^. *n*_↑_ (*n*_↓_) is the carrier density of spin up (down) at zero-field, and *f*_↑_ (*f*_↓_) is the SdH oscillation frequency corresponding to spin up (down). The spin-splitting according to the Rashba Hamiltonian gives the following relation for the spin-orbit coupling constant, ${\alpha }_{R}=\,\frac{{{\rm{\Delta }}}_{R}}{2{k}_{F}}$^40^. Fig. 5(a) shows *α*_*R*_ of WS_2_/BLG/WS_2_ sandwich devices as a function of gate voltages. V_bg_ effectively changes *α*_*R*_ for V_bg_ < 0, whereas *α*_*R*_ does not change much for V_bg_ > 0 due to the screening effect of the WS_2_ layer. The zero-field spin-splitting ${{\rm{\Delta }}}_{R}\,\,$is plotted as a function of V_bg_ at fixed V_tg_ in Fig. 5(b). The estimated value of Δ_R_ in our device is much stronger than the theoretically predicted value of graphene on conventional substrate^41^.

If the spin relaxation mechanism is dominated by the Dyakonov-Perel mechanism based on Rashba type spin-orbital interaction, the following relation holds: ${\tau }_{so}=\,\frac{{\hslash }^{4}}{4{\alpha }_{so\,}^{2}D\,{m}^{\ast 2}}.$^23,42,43^ By using this relation, we calculated α_so_ from *τ*_*so*_ that was obtained as fitting parameters of WAL theory in Fig. 3(a) and (b). The SOI-induced spin-splitting can be obtained by using the following relation $:\,{{\rm{\Delta }}}_{SO}\,=2{k}_{F}{\alpha }_{so}$. Figure 5(c) shows ${{\rm{\Delta }}}_{SO}\,\,$as a function of V_bg_ at V_tg_ = 0 V, along with $\,{{\rm{\Delta }}}_{R}$. Figure 5(d) shows ${{\rm{\Delta }}}_{SO}\,\,$and $\,{{\rm{\Delta }}}_{R}$ as a function of V_tg_ at V_bg_ = −35 V. Remarkably, ${{\rm{\Delta }}}_{SO}$ and ${{\rm{\Delta }}}_{R}\,\,$have the same trend with approximately close values. Spin-splitting estimated by the two different ways eventually give similar values, supporting the self-consistency of our experiments.

**Figure 6:**
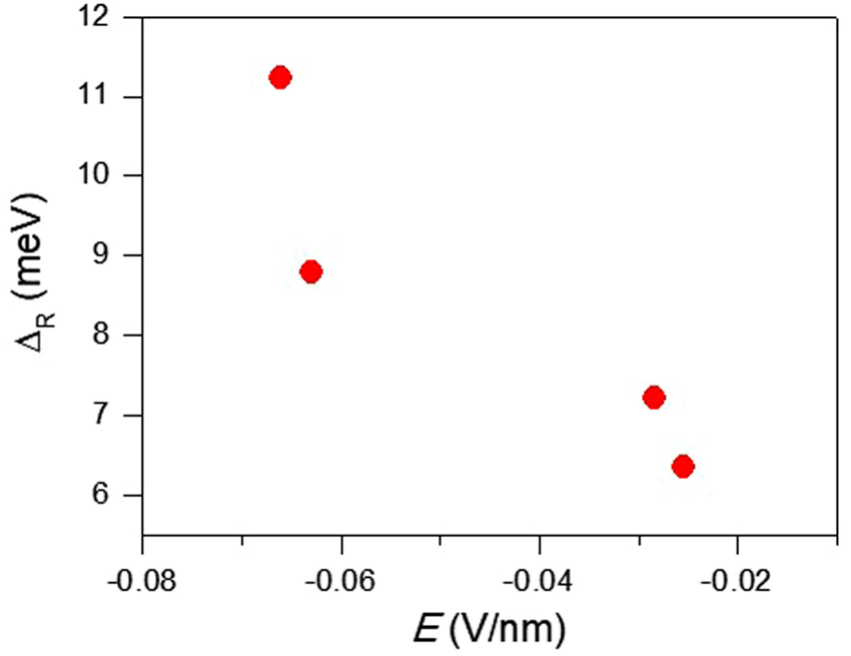
Rashba spin-splitting (${{\rm{\Delta }}}_{R}$) as a function of gate electric field (*E*) at T= 4.2 K. The average hole density is 1.91 × 10^12^ cm^−2^.

One of the advantages of dual gate configuration is that it is possible to tune the carrier density and electric field independently. It is worthwhile to clarify that the Rashba type SOI can be modified by the external applied electric field without changing the carrier density (*n*). At first we have estimated *n* by Hall measurements at all combination of transverse electric fields and have selected a group of gate voltages at which *n* is approximately same ($\frac{{\rm{\Delta }}n}{n} < 15\, \%$). We have chosen a group with average hole density, *n* = 1.91×10^12^ cm^−2^. Further, we have calculated the gate electrical field (*E*) by using the relation, *E* = (*V*_bg_ – *V*_tg_)/*d*, where *d* is the effective thickness depending on the dielectric constants (ε) of the materials between top and back-gate electrodes. The effective thickness is given by ${\rm{d}}={d}_{Si{O}_{2}}\frac{{\varepsilon }_{W{S}_{2}}}{{\varepsilon }_{Si{O}_{2}}}+{d}_{W{S}_{2}}+{d}_{A{l}_{2}{O}_{3}}\frac{{\varepsilon }_{W{S}_{2}}}{{\varepsilon }_{A{l}_{2}{O}_{3}}}$, where ${\varepsilon }_{Si{O}_{2}}$ = 3.9, ${\varepsilon }_{W{S}_{2}}$ = 6.5, ${\varepsilon }_{A{l}_{2}{O}_{3}}$ = 9.8, and *d*_*i*_ is the thickness of SiO_2_, WS_2_ and Al_2_O_3_, respectively. Then we have plotted the Rashba spin-splitting (${{\rm{\Delta }}}_{R}$) as a function of *E* in Fig. 6. We have found that ${{\rm{\Delta }}}_{R}$ is enhanced with the applied electric field. It has been demonstrated that the Rashba type SOI is enhanced by the external electric field without changing of the carrier density.

In summary, a dual gated WS_2_/BLG/WS_2_ sandwich structure to investigate the modulation of SOI due to interfacial build-up potentials was fabricated in this study. The dual gate enabled an effective control of the charge carrier density and transverse electric field, which broke the inversion symmetry of BLG. The dominant part of the interface-induced SOI was Rashba type, which can be confirmed by SdH oscillations. One auspicious objective was to enhance the SOI in BLG, as this may offer several possibilities including the manipulation of spin current through an electric field or the generation of a pure spin current through the Spin Hall effect. By placing BLG between WS_2,_ there was an enhancement in the mobility as high as ~20,000 cm^2^/Vs. The WAL effect was found in the magneto-conductivity at T= 4.2 K. τ_so_ was also obtained by fitting the magneto-conductivity to the WAL theory. A strong enhancement of SOI was confirmed by the estimated τ_so_ of BLG encapsulated by WS_2_ layers. The spin-orbit coupling constant was deduced from two different measurements: the WAL effect and SdH oscillations. The estimated spin-orbit coupling constant showed close values as a function of gate voltages. The SOI-induced spin-splitting changed from 5 to 25 meV depending on gate voltages. Given that the spin-orbit coupling constant was controlled effectively by gate voltage, the WS_2_/BLG/WS_2_ sandwich structure should be a strong candidate as a channel material for a spin field effect transistor^44^.

## Methods

### Device fabrication

Multilayer WS_2_ flake was exfoliated on SiO_2_/highly doped p-type Si (300 nm) substrates acting as a back gate by using the standard Scotch tape method. Bilayer graphene (BLG) was transferred onto 7-nm-thick WS_2_ films by a dry transfer method. In order to transfer BLG onto WS_2_, a thin film of polymer (polyvinyl alcohol, PVA) was coated on Si wafer by spin-coating. PVA acted as a water-soluble layer, and a poly (methyl methacrylate) (PMMA, 495 A-6) layer was formed on top as a supporting layer for graphene by spin-coating. On top of the PMMA, graphene was exfoliated from commercial graphite using the standard adhesive tape method. This study employed an optical microscope to estimate the rough thickness of BLG flake. Graphene with bilayer thickness can be identified in the PMMA layer with appropriate thickness due to the interference effect. The thickness of BLG was further confirmed by Raman spectroscopy. The PVA layer was dissolved by deionized water (DI), and the PMMA membrane subsequently floated on the water surface. The BLG flake was transferred onto WS_2_ flake in a big pattern^45^ by using a micro-aligner stage and examined by a high-resolution camera. After the transfer, the sample was annealed at 200 °C for 6 hours under Ar/H_2_ (97.5% Ar/2.5% H_2_) gas flow. The uncovered part of WS_2_ was covered by 20-nm-thick Al_2_O_3_ by atomic layer deposition (ALD) to prevent direct contact of the electrode with WS_2_. The electrodes were designed by electron-beam lithography, and Cr/Au (6/60 nm) was deposited by thermal evaporation. The length and width of the BLG channel were 3 μm and 1.1 μm, respectively. The top surfaces of Cr/Au electrodes were covered by Al_2_O_3_ by ALD. The top 10-nm-thick WS_2_ film was transferred by a dry method with the help of polydimethylsiloxane (PDMS). Finally, a top gate was fabricated on WS_2_ after 30-nm-thick Al_2_O_3_ deposition. To clean the device, it was annealed in a tube furnace at a temperature of 200 °C under Ar/H_2_ (97.5% Ar/2.5% H_2_) gas flow for 4 h^27,46^.

### Device characterization and transport measurement

The Raman spectra of both BLG and WS_2_ were measured with a Renishaw micro spectrometer over a wave number range of 1100 to 3200 cm^−1^ in the case of BLG and 200 to 500 cm^−1^ for WS_2_, with a laser wavelength of 514.5 nm. The spot size was 1 μm, and the power was kept at 1.0 mW to prevent the device from experiencing local heating. An atomic force microscope (AFM) was used to examine the surface morphology of BLG and WS_2_. The magnetotransport measurements of the WS_2_/BLG/WS_2_ sandwich devices were performed by using the standard lock-in technique at low temperature in a cryostat while perpendicular magnetic field was applied.

## Electronic supplementary material

Supplementary Information
