# Supplementary material for: Gate Modulation of the Spin-orbit Interaction in Bilayer Graphene Encapsulated by WS2 films
Source: Sci Rep. 2018 Feb 21;8:3412. doi: 10.1038/s41598-018-21787-y (PMC5821884; doi:10.1038/s41598-018-21787-y)
Supplement: Supplementary file 1 — Supplementary Information [file 41598_2018_21787_MOESM1_ESM.pdf]

Supplementary Information

Gate Modulation of the Spin-orbit Interaction in Bilayer Graphene Encapsulated by WS_2 films

Amir Muhammad Afzal[1], Muhammad Farooq Khan[1], Ghazanfar Nazir[1], Ghulam Dastgeer[1], Sikandar Aftab[1], Imtisal Akhtar[2], Yongho Seo[2] and Jonghwa Eom[1*]

[1]Department of Physics & Astronomy and Graphene Research Institute, Sejong University, Seoul 05006, Korea

[2]Department of Nanotechnology & Advanced Materials Engineering, Sejong University, Seoul 05006, Korea

*E mail: eom@sejong.ac.kr

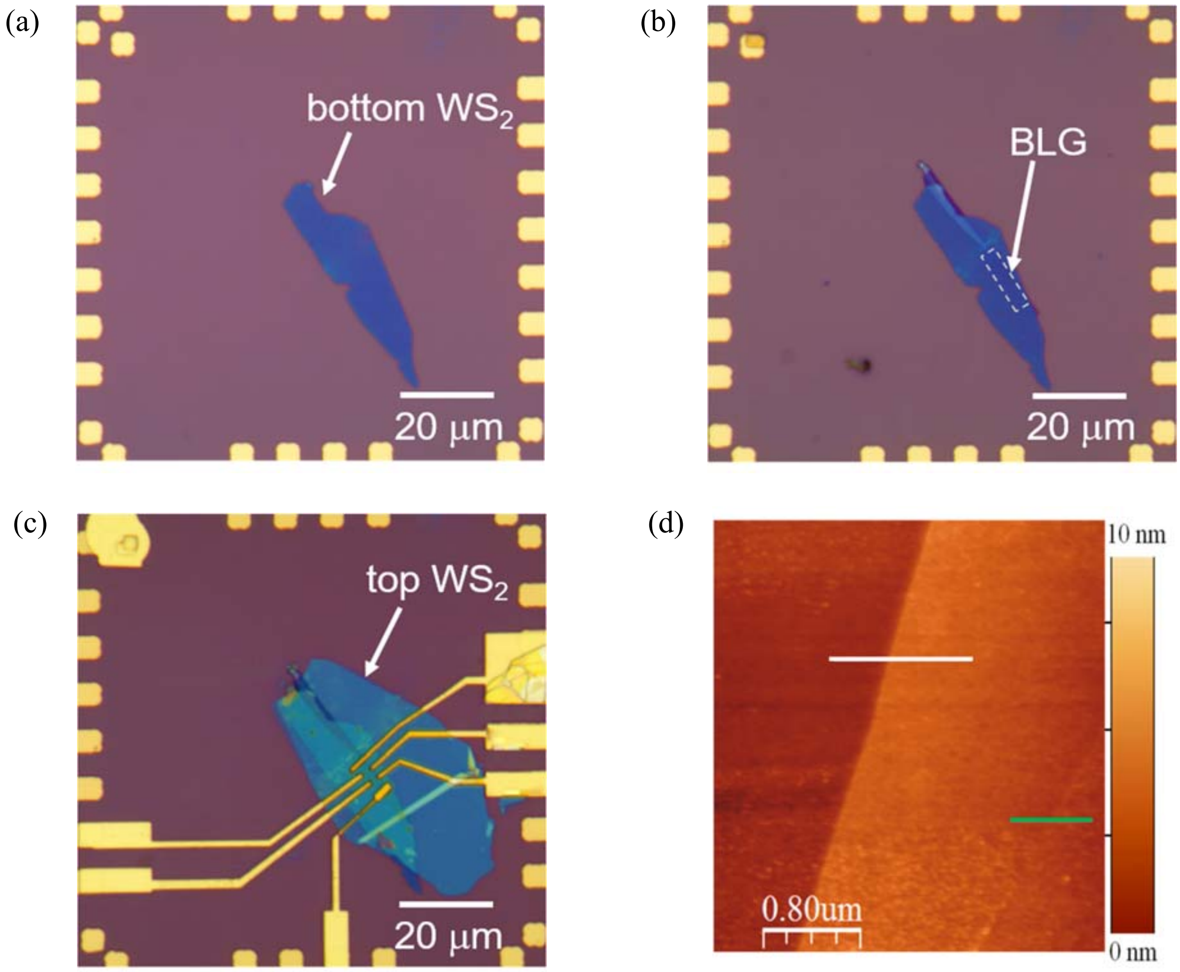

Figure S1. Complete device fabrication of WS_2/BLG/WS_2 heterostructure device (a) WS_2 flake on SiO_2 substrate. (b) Graphene is transferred by using polyvinyl alcohol (PVA) on WS_2 flake, and then the naked part of WS_2 is covered with 20-nm-thick Al_2O_3 by the atomic layer deposition (ALD) method. (c) Complete WS_2/BLG/WS_2 heterostructure device. Final electrodes are made by E-beam lithography (8/60-nm-thick Cr/Au). (d) Atomic force microscope (AFM) image of the device showing WS_2 on SiO_2 and BLG on WS_2. The white and green lines represent the scanning lines for the height profile of WS_2 on SiO_2 and BLG on WS_2 in Figure 2(c) and (d).

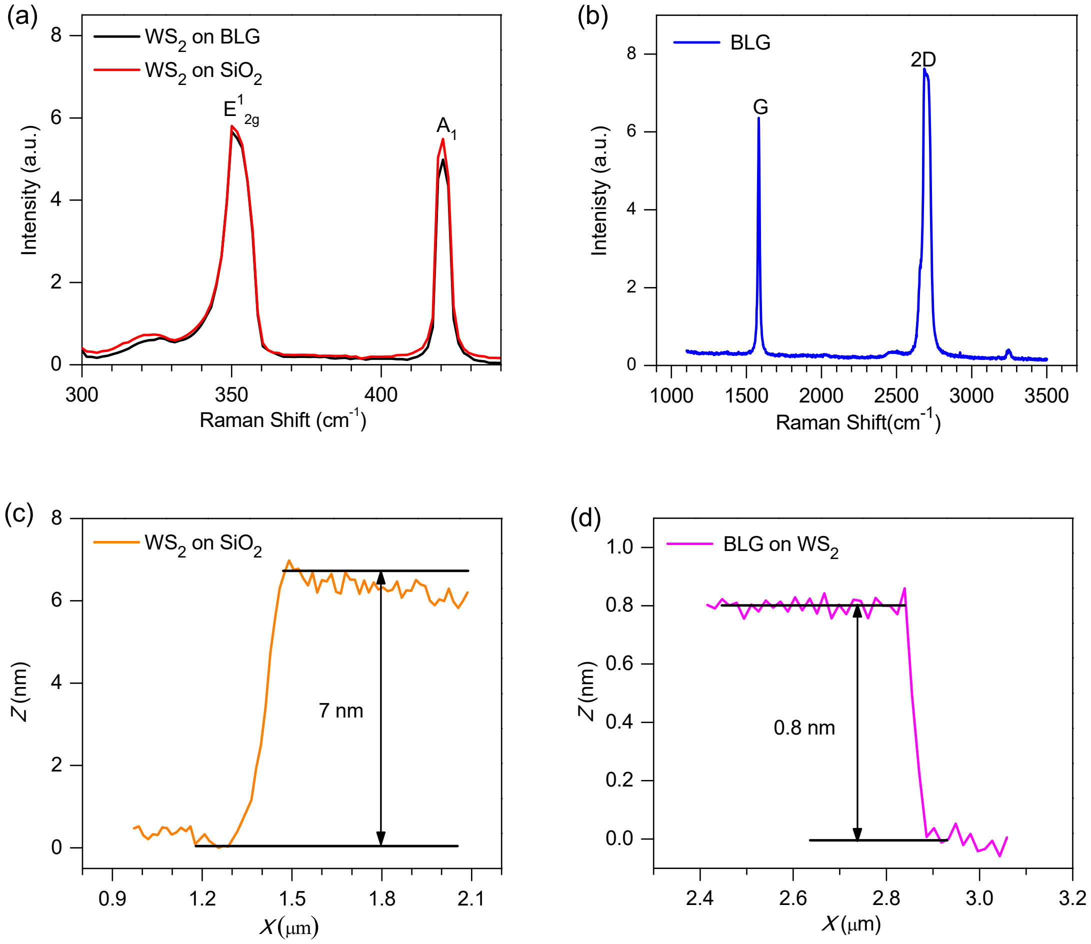

Figure S2. Raman spectrum and atomic force microscopy (a) Raman spectrum of multilayers of WS_2 with or without graphene. The peaks appear at 351 cm^{-1} and 418 cm^{-1}. The intensity of the 418 cm^{-1} peak slightly decreases on graphene (Gr). (b) Raman spectrum of BLG. The ratio of intensities of G and 2D peaks (I_{2D}/I_G) is ~1.2, which is consistent with the previously reported values of BLG. (c) The height profile of WS_2 on SiO_2. The thickness of WS_2 is ~7 nm. (d) The height profile of BLG on WS_2. The thickness of BLG flake is ~0.8 nm.

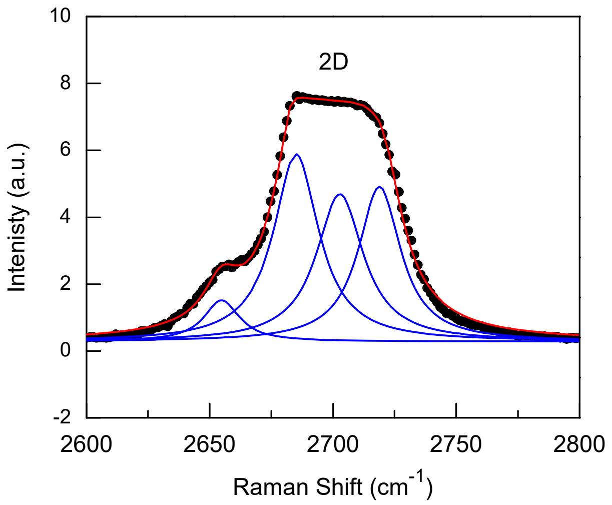

Figure S3. Raman spectrum of bilayer graphene (BLG) on WS_2 flake. 2D peak is fitted by the sum of four Lorentz peaks, which confirms the sign of BLG.

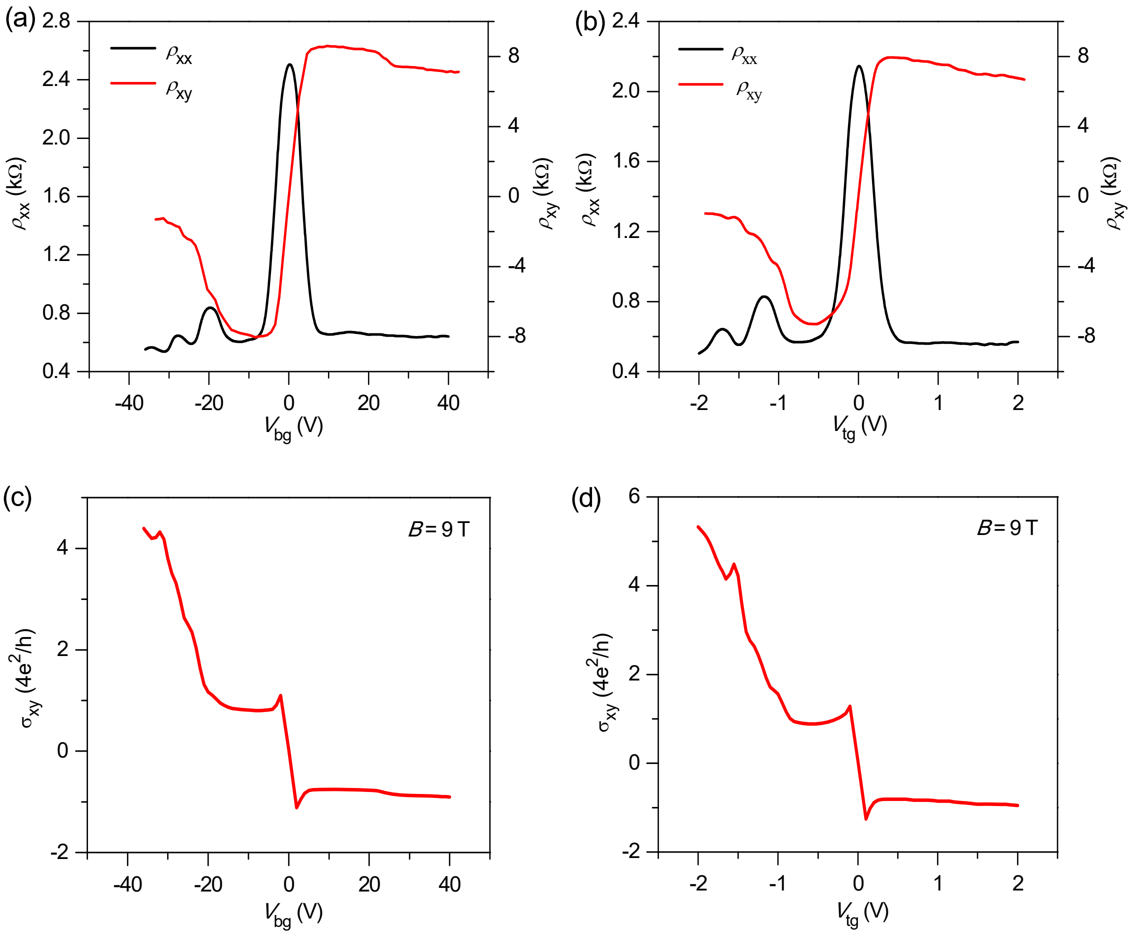

Figure S4. Magnetotransport measurement in high magnetic field in the WS_2/BLG/WS_2 heterostructure device (a) Longitudinal and Hall resistivity as a function of V_{bg} at a fixed magnetic field of 9 T at 4.2 K. ρ_{xx} and ρ_{xy} do not change much for gate voltages above V_{th} of WS_2 due to the screening effect. (b) Longitudinal and Hall resistivity as a function of V_{tg} at a fixed magnetic field of 9 T at T= 4.2 K. (c) Hall conductivity as a function of V_{bg}. (d) Hall conductivity as a function of V_{tg}.
